# Multisystem Inflammatory Syndrome Temporally Related to COVID-19 in Children From Latin America and the Caribbean Region: A Systematic Review With a Meta-Analysis of Data From Regional Surveillance Systems

**DOI:** 10.3389/fped.2022.881765

**Published:** 2022-04-25

**Authors:** Silvina Ruvinsky, Carla Voto, Macarena Roel, Ana Fustiñana, Natalia Veliz, Martin Brizuela, Susana Rodriguez, Rolando Ulloa-Gutierrez, Ariel Bardach

**Affiliations:** ^1^Coordinación de Investigación Clínica y Sanitaria, Hospital de Pediatría “Prof. Dr. Juan P. Garrahan”, Ciudad Autónoma de Buenos Aires, Argentina; ^2^Servicio de Emergencias, Hospital de Pediatría “Prof. Dr. Juan P. Garrahan”, Ciudad Autónoma de Buenos Aires, Argentina; ^3^Área de Internación, Hospital de Pediatría “Prof. Dr. Juan P. Garrahan”, Ciudad Autónoma de Buenos Aires, Argentina; ^4^Hospital General de Agudos “Vélez Sarsfield”, Ciudad Autónoma de Buenos Aires, Argentina; ^5^Servicio de Infectología, Hospital Nacional de Niños Dr. Carlos Sáenz Herrrera, Caja Costarricense de Seguro Social & Universidad de Ciencias Médicas (UCIMED), San José, Costa Rica; ^6^Center for Research in Epidemiology and Public Health, Institute for Clinical Effectiveness and Health Policy (IECS) and National Scientific and Technical Research Council (CONICET), Buenos Aires, Argentina

**Keywords:** MIS-C, COVID-19, SARS-CoV2, children and adolescents, prevalence, prognosis, use of resources

## Abstract

**Background:**

With the emergence of the COVID-19 pandemic, increasing numbers of cases of the multisystem inflammatory syndrome in children (MIS-C) have been reported worldwide; however, it is unclear whether this syndrome has a differential pattern in children from Latin America and the Caribbean (LAC). We conducted a systematic review and meta-analysis to analyze the epidemiological, clinical, and outcome characteristics of patients with MIS-C in LAC countries.

**Methods:**

A systematic literature search was conducted in the main electronic databases and scientific meetings from March 1, 2020, to June 30, 2021. Available reports on epidemiological surveillance of countries in the region during the same period were analyzed.

**Results:**

Of the 464 relevant studies identified, 23 were included with 592 patients with MIS-C from LAC. Mean age was 6.6 years (IQR, 6–7.4 years); 60% were male. The most common clinical manifestations were fever, rash, and conjunctival injection; 59% showed Kawasaki disease. Pool proportion of shock was 52%. A total of 47% of patients were admitted to the pediatric intensive care unit (PICU), 23% required mechanical ventilation, and 74% required vasoactive drugs. Intravenous gamma globulin alone was administered in 87% of patients, and in combination with steroids in 60% of cases. Length of hospital stay was 10 days (IQR, 9–10) and PICU stay 5.75 (IQR, 5–6). Overall case fatality ratio was 4% and for those hospitalized in the PICU it was 7%.

**Conclusion:**

Limited information was available on the clinical outcomes. Improvements in the surveillance system are required to obtain a better epidemiologic overview in the region.

## Introduction

COVID-19 is usually mild in pediatric patients (2). Children presenting with severe shock-like syndrome, incomplete Kawasaki disease (KD), or toxic shock syndrome (3) were reported in the United Kingdom and Italy in April 2020. Subsequently, children with a similar clinical presentation were also reported in the rest of Europe, America, and South Africa (4). The entity was identified as multisystem inflammatory syndrome in children (MIS-C) associated with SARS-CoV-2 infection with a spectrum of manifestations, such as KD, toxic shock syndrome, sepsis, and macrophage activation syndrome.

The World Health Organization (WHO) (5), the Center for Disease Prevention and Control (CDC) (6), and the Royal College of Pediatrics and Child Health of the United Kingdom (RCPCH) (7) issued definitions for case identification. Systematic reviews based on case reports, case series, and observational studies have been published; however, these include children from different backgrounds and ethnicities, mainly from Europe and North America. MIS-C appears to vary between regions, with few cases reported in children from Asia (8). Poor accessibility to the health system and delay in diagnosis and treatment in countries with limited resources may lead to a poor prognosis in children with MIS-C in Latin America and the Caribbean region (LAC). Some research has been conducted in LAC, but the topic is largely unexplored. Existing data come from specific collaborative networks of intensive therapists, cardiologists, rheumatologists, and pediatric infectious disease practitioners in the region, but these findings have not been routinely collected or analyzed. To our knowledge, there are no published systematic reviews that include surveillance data and epidemiological records about MIS-C in Latin American children.

The current study aimed to describe the clinical course, laboratory findings, epidemiology, treatment, and use of resources of MIS-C in children from LAC through a systematic review and a meta-analysis incorporating data from regional surveillance systems.

## Materials and Methods

For this systematic review and meta-analysis, we followed the Cochrane methods and the 2020 Preferred Reporting Items for Systematic Reviews and Meta-Analyses (PRISMA) (9) statement for reporting results. The protocol for the present systematic review was registered (10) in the University of York’s PROSPERO database (CRD42021242505).

Risk of bias was assessed by two reviewers. Disagreements were resolved by consensus of the entire team. If consensus could not be reached, the conflict was resolved by a third reviewer.

For cohort and cross-sectional studies, the NIH instrument was used to score 14 items. Six studies were assessed as fair quality and three as good quality, with a score as having a moderate-to-low risk of bias. The best scores were for research questions and population selection. On the other hand, the included case series had a quality assessment as poor in five, fair in seven and good quality in one, with a high risk of bias. The only included case-control study received a fair quality score with a moderate-to-high risk of bias. In summary, most of the included studies were rated as being of low-to-moderate methodological quality with a moderate-to-high risk of bias. Risk of bias is shown in Supplementary Tables 2–4.

We applied an arc-sine transformation to stabilize the variance of proportions following the Freeman-Tukey variant of the arc-sine square root of transformed proportions method (11).

### Eligibility Criteria

Comparative and non-comparative study designs were included regardless of publication status, publication year, or language. Studies without a clear denominator, narrative and case series reviews, and articles with unavailable full text and systematic reviews (SRs) were excluded; however, the reference lists of SRs on the subject were examined for relevant studies.

We searched the Latin-American and Caribbean Health Sciences Literature (LILACS), Medline, Embase, SciELO, Cochrane Library database, and WHO Database publications on MIS-C and SARS-CoV-2 and CRD York Prospero and preprint databases (ArXiv, BiorXiv, medRxiv, search.bioPreprint). We also searched the proceedings of international, national, or regional (LAC) scientific meetings from March 1st, 2020, to June 30th, 2021. The search strategies utilized can be found in Supplementary Material.

### Outcomes of Interest

We explored epidemiological outcomes (prevalence, incidence, mortality), use of resources, clinical outcomes and complications, laboratory findings, and overlap with KD.

### Study Selection, Data Extraction, and Assessment of the Risk of Bias in Included Studies

Pairs of reviewers independently screened articles for selection, evaluating titles and abstracts of studies. Discrepancies were solved by consensus of the whole team. We used COVIDENCE Software (12) for the initial phases of the systematic review. We also explored the reports of passive surveillance report systems from Pan American Health Organization (PAHO) and LAC countries.

Potentially eligible studies were retrieved in full text, and two reviewers independently extracted and assessed the risk of bias. Disagreements were also resolved by discussion among the review team members. For data extraction an online spreadsheet was used. This was piloted initially on ten papers to refine the process. The research team extracted study characteristics (type of publication, year published, authors, geographic location, study design including risk of bias method), population characteristics, and outcomes (incidence rate, specific mortality, and fatality rate). Authors of articles were contacted when necessary for Supplementary Information.

The risk of bias of observational studies and the control arm of trials was assessed using a checklist developed by the United States National Heart, Lung, and Blood Institute (13), which classifies studies as high (Poor), moderate (Fair), and low (Good) risk of bias. Cross-sectional and case series require 14 items, while cohort studies require nine items and case-control studies require 12 items.

### Data Synthesis

To analyze the data, proportion meta-analyses were conducted. We applied arc-sine transformation to stabilize the variance of proportions (Freeman-Tukey variant of the arc-sine square-root of transformed proportions method), y = arcsine[√(r/(n + 1))] + arcsine[√(r/(n + 1)/(n + 1)], with variance of 1/(n + 1), where n is the population size. Pooled proportions were calculated as the back-transformation of the weighted mean of the transformed proportions, using inverse arcsine variance weights for the fixed and random-effects model. We applied DerSimonian-Laird weights for the random-effects model where heterogeneity between studies was found. We calculated the I^2^ statistic to measure the proportion of the overall variation attributable to between-study heterogeneity. The R software package *meta* and its functions *metamean*, *metaprop*, and *forest.meta*, and STATA 14.0 were used. Extracted data were synthesized using both descriptive and meta-analytic approaches by outcome measures (RRs or Mantel-Haenszel ORs) or (Peto ORs) for dichotomous data. Mean difference (MD) and 95% confidence interval (CI) were reported for all outcomes for continuous data. We used simple descriptive statistics whenever impossible to calculate association measurements.

## Results

**FIGURE 1:**
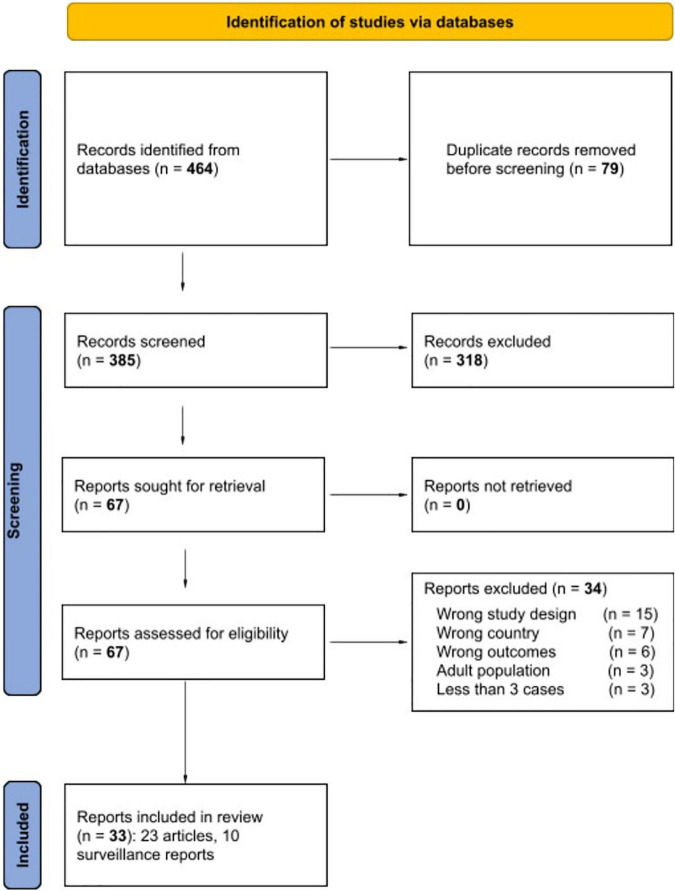
PRISMA 2020 study flow diagram. The PRISMA 2020 checklist and the evidence quality scores of the articles included (risk of bias) can be found in Supplementary Table 1.

The search strategy yielded 464 potentially relevant studies in the databases. Figure 1 shows a diagram of the study selection process. Twenty-three full-text studies that met the inclusion criteria for data synthesis were included. All were observational studies with 13 case series, seven cohort studies, two cross-sectional studies, and one case-control study. In addition, surveillance system reports were included for separate analysis. The most frequent reasons for exclusion were wrong study design (*N* = 15), wrong country (*N* = 7), wrong outcomes (*N* = 6), adult population (*N* = 3), and three studies were left out because they reported fewer than three cases. The included studies collected data from 592 patients from Peru, Brazil, Argentina, Chile, Costa Rica, Colombia, Mexico, Venezuela, and other countries. The main characteristics of clinical studies of MIS-C included are described in Table 1. The mean age reported was 6.6 years (IQR, 6–7.4 years); 355 (60%) were male (95% CI, 55–65). A list of excluded studies during the full text assessment can be found in Supplementary Table 5 (65–67).

**TABLE 1:** Characteristics of the clinical studies of MIS-C identified in Latin America and the Caribbean (*N* = 23).

References	Country	Outcomes	Number of patients	Study design	Time period	Age (years) Mean (*SD*)	Male/ Female
Coronado Muñoz et al. (14)	Peru	Clinical manifestations (mortality during hospitalization)	21	Cohort	March-August, 2020	7 (5.4)	15/6
Seery et al. (15)	Argentina	Laboratory features	21	Cohort	May-Oct, 2020	6 (5.9)	16/5
Coila Paricahua et al. (16)	Peru	Use of resources	13	Case series	April-Oct, 2020	8 (2.6)	7/6
Torres et al. (17)	Chile	Clinical manifestations, laboratory features, complementary studies, and use of resources	27	Cohort	May-June, 2020	6*	14/13
De Coll-Vela et al. (18)	Peru	Clinical manifestations, laboratory features, complementary studies, and treatment	8	Case series	May-June, 2020	5.5*	5/3
Sandoval et al. (19)	Chile	Clinical manifestations (neurologic manifestations)	17	Case series	April–July, 2020	6.5*	NR
Fontes (20)	Brazil	Clinical manifestations	42	Case series	July–Dec, 2020	8*	25/17
Lima-Setta et al. (21)	Brazil	Clinical manifestations, laboratory features, complementary studies, and treatment	56	Cohort	March–July, 2020	6.2 (5.9)	39/17
del Aguila et al. (22)	Peru	Clinical manifestations (Kawasaki, shock), use of resources (ICU, ventilation)	37	Case series	Apri–August, 2020	8 (4.4)	25/12
Álvarez et al. (23)	Chile	Clinical manifestations (Kawasaki, shock), use of resources (ICU)	23	Case series	May-July, 2020	6.2*	14/9
Niño-Taravilla et al. (24)	Chile, Colombia, Peru, Others	Use of resources (mechanical ventilation, inotropic support, complementary studies)	26	Case series	May-August, 2020	6.5 (6.3)	15/11
Ivankovich-Escoto et al. (25)	Costa Rica	Clinical manifestations	11	Case series	NR	5.4 (2.1)	7/4
Brenes-Chacón et al. (26)	Costa Rica	Clinical manifestations	26	Case series	March 2020–January 2021	6.2*	12/14
Gutiérrez-Hidalgo et al. (27)	Costa Rica	Clinical manifestations, use of resources	4	Case series	NR	7 (4.9)	2/2
Luna-Muñoz et al. (28)	Peru	Clinical manifestations, use of resources	10	Case series	June-August, 2020	7 (2.4)	7/3
de Farias et al. (29)	Brazil	Clinical manifestations, laboratory features, complementary studies, and use of resources	11	Case series	April–June, 2020	4.9 (3)	9/2
Duarte-Neto et al. (30)	Brazil	Clinical manifestations, laboratory features, complementary studies, and treatment	3	Case series	March–August, 2020	8 (2.2)	1/2
Antúnez-Montes et al. (31)	Mexico, Colombia, Peru, Costa Rica, Brazil	Clinical manifestations, complementary studies, treatment, and use of resources	95	Cohort	June–August, 2020	7*	52/43
Rosanova et al. (32)	Argentina	Clinical manifestations, laboratory features, complementary studies and treatment	25	Case control	April–October, 2020	8.7*	9/16
Prata-Barbosa et al. (33)	Brazil	Clinical manifestations, laboratory features, complementary studies	10	Cohort	March–May, 2020	5.2 (5.1)	8/2
Yock-Corrales et al. (34)	Argentina, Colombia, Costa Rica, Mexico, Peru	Use of resources (antibiotic use)	69	Cohort	April–October, 2020	6 (3.5)	45/24
Pereira et al. (35)	Brazil	Clinical manifestations, laboratory features and use of resources	6	Cross-sectional	April–June, 2020	7.8*	5/1
Domínguez Rojas et al. (36)	Peru	Clinical manifestations and treatment	31	Cross-sectional	March–August, 2020	5.4*	18/13

*SD not reported; NR, Not reported.

### Clinical Characteristics of the Study Population

A total of 23 studies with 592 patients with confirmed MIS-C associated with SARS-CoV-2 infection were included. Of the 23 studies, 11 mentioned following the CDC criteria for MIS-C definition in at least some of the included patients (*n* = 291), six followed the WHO criteria (110 patients), and one the RCPCH criteria (37 patients). One study with a total of 26 patients used the criteria by the Public Health Ministry of Chile. Most studies were case series (Table 1).

The principal outcomes analyzed were laboratory features, clinical manifestations and outcome, complementary studies, treatment, and use of resources. In most of the studies included, the male sex was predominant (Table 1).

### Clinical Manifestations

Most patients reported were previously healthy, while 32% had a history of one or more comorbidities, most frequently obesity, diabetes, chronic pulmonary disease, heart disease, immunocompromised, neurologic disease, and liver or kidney disease. Near half of the patients had contact with a confirmed SARS-CoV-2-infection case. One-third of the patients analyzed had positive RT-PCR SARS-CoV-2 tests, and 74% had positive SARS-CoV-2 serology tests (Table 2). The most frequent clinical manifestations were fever, rash, and conjunctival injection. KD was reported in 12 studies with a total of 161 of 273 patients (pool proportion 61%; 95% CI, 48–79%). Shock was present in 142 of 331 with a pool proportion of 52% (Table 2 and Supplementary Table 6).

**TABLE 2:** Clinical characteristics, laboratory features, and use of resources: meta-analyses.

Variables	Studies (*), n/N	Cases/Total, n/N	Pooled proportion [95% CI] from meta-analyses
Age, years (median, IQR)	23/23	592/592	6.5 years (6–7.4 years)
Male sex	23/23	355/592	0.60 [0.55–0.65]
Previously healthy	16/23	250/433	0.71 [0.52–0.84]
Obesity	9/23	18/179	0.11 [0.06–0.20]
Close contact	14/23	153/317	0.59 [0.39–0.76]
Positive PCR	18/23	139/449	0.32 [0.22–0.44]
Positive serology	20/23	302/472	0.74 [0.58–0.86]
Fever	18/23	378/423	0.99 [0.9–1]
Rash	11/23	167/258	0.74 [0.51–0.89]
Conjunctival injection	11/23	149/264	0.67 [0.42–0.86]
Kawasaki disease (KD)	12/23	161/273	0.61 [0.48–0.79]
Shock	16/23	142/331	0.52 [0.34–0.70]

(*) (14–36).

Fifteen studies reported patients with one or more gastrointestinal symptoms; diarrhea was the most common (221 patients, 60%), followed by abdominal pain (175 patients, 47%), and vomiting (144 patients, 38%). Respiratory symptoms were reported in 16 studies with 372 patients; cough was the most common symptom (94 patients, 25%), followed by dyspnea (42 patients, 11%).

In 11/23 studies neurological manifestations were reported, of which headache was the most common (82 patients, 23%). Eleven studies reported mucocutaneous symptoms, most commonly rash and conjunctival injections, followed by edema (45 patients, 17%) and lymphadenopathy (15 patients, 6%).

Inflammatory status was confirmed by laboratory tests in most studies (Table 3 and Supplementary Figure 1). CRP and D-dimer were elevated. Lymphopenia was observed in most patients. A slight decrease of albumin level was also reported.

**TABLE 3:** Laboratory findings, pooled results: meta-analyses.

	Studies (*), *n*/N	Random effects (Mean [95% CI])
**C-reactive protein (CRP)**	12/23	19.8 [14.27–27.54] mg/dL
**Ferritin**	11/23	394.46 [294.6–528] ng/mL
**D-dimer**	11/23	3,275 [2,504–4,285] ng/mL
**Lymphocyte count**	5/23	1,196 [1,087–1,316] cell/mm3
**Platelets**	9/23	182,745 [172,931–193,116] cell/mm3
**Albumin**	7/23	2.83 [2.64–3.02] g/dL

(*) (14–36).

### Cardiac Involvement in Patients With and Without Kawasaki Disease

Of the total 273 patients included, 59% (*N* = 161) were patients meeting KD criteria. In these reports, 32% (*N* = 87 patients) had abnormal echocardiograms, 21% (*N* = 59 patients) had pericarditis or pericardial effusion, 12.8% (*N* = 35) ventricular dysfunction, 14% (*N* = 38) coronary aneurysm/dilatation/abnormality, 8% (*N* = 23) myocarditis, 2,5% (*N* = 7) valvular dysfunction, 4% (*N* = 12) abnormal ECG, while 15% (*N* = 41) of the patients had elevated BNP and troponin levels.

A total of 11 reports analyzed cardiac involvement in 319 patients without KD; 8% (*N* = 28) had abnormal echocardiograms, 3% (*N* = 11) pericarditis or pericardial effusion, 2.5% (*N* = 8) myocarditis, 2% (*N* = 7) ventricular dysfunction, 4% (*N* = 13) coronary aneurysm/dilatation/abnormality, without valvular dysfunction or abnormal ECG, 1.6% (*N* = 5) had elevated BNP levels and 1.2% (*N* = 4) had elevated troponin levels (Supplementary Table 7).

### Treatment and Use of Resource

**FIGURE 2:**
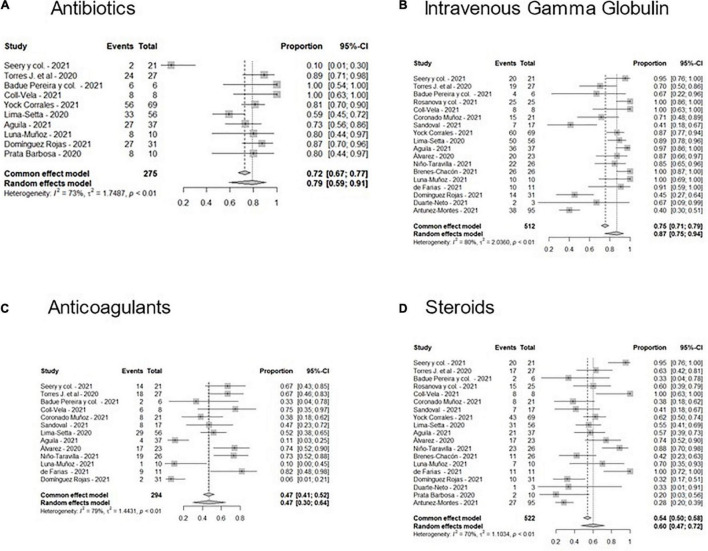
Forest plots of the frequencies of treatments administered in MIS-C patients in Latin America. **(A)** Antibiotics, **(B)** Intravenous Gamma Globulin, **(C)** Anticoagulants, and **(D)** Steroids.

Figure 2 shows the Forest plots with meta-analyses of the different treatments administered. Most patients received antibiotics and gamma globulin alone or in combination with corticosteroids; only seven patients received tocilizumab or siltuximab. A pool of 47% of the patients received anticoagulants.

**FIGURE 3:**
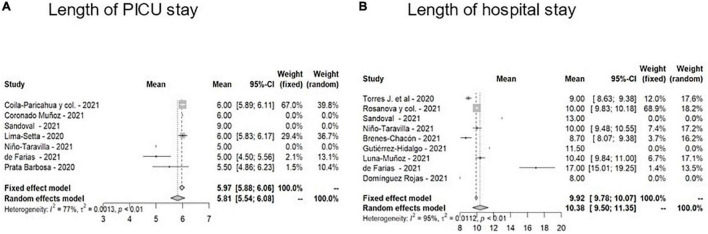
Forest plots of length of stay of patients in the PICU **(A)** or on the general ward **(B)**.

As regards the use of resources (Figure 3) in 7/23 studies, median PICU stay reported was 5.75 (IQR, 5–6 days), and in 9/23 studies, median hospital stay was 10 days (IQR 9–10). Overall case fatality rate observed was 4% (25/592).

### Clinical, Laboratory, and Outcome of Multisystem Inflammatory Syndrome in Children in the Subgroup of Patients Admitted to the Pediatric Intensive Care Unit

In fifteen studies with a total of 415 patients, need for PICU admission was reported in 47% (195 patients). Six studies described severe MIS-C requiring intensive care; four studies were from Brazil, one from Peru, and the remaining one from Chile. Severe MIS-C occurred in males in 71% of cases and 34% had one or more comorbidities. Fever was observed in 98% of children and cardiovascular impairment in 85%. Mechanical ventilation was required in 23%. In only four studies, vasoactive drugs were used in 74% of the children. Most patients were treated with IVIG (88%), and 70% received steroids, 68% aspirin, and 58% anticoagulation. The overall mortality rate in patients with MIS-C admitted to the PICU was 7%. Clinical features, laboratory characteristics, and resource utilization in patients requiring PICU are described in Table 4 and in Supplementary Tables 8–10.

**TABLE 4:** Pooled meta-analyses in MIS-C patients admitted to the PICU.

	Studies, *n*/*N* (*)	Random effects (Mean [95% CI])
**Age**	5/5	5.15 [5.01–6.61] years
**Male**	5/5	73% [63–82%]
**Platelets**	4/5	145,344 [115,027–183,652] cell/mm3
**C-reactive protein (CRP)**	5/5	25.9 [10.76–62.37] mg/dL
**Ferritin**	5/5	469.99 [372.52–592.97] ng/mL
**D-dimer**	5/5	3465.55 [3,031–3,961] ng/mL
**Non-invasive ventilation**	3/5	12% [4–33%]
**Mechanical ventilation**	5/5	36% [11–70%]
**Inotropes**	3/5	73% [55–85%]
**IVIG**	4/5	88% [79–93%]
**Steroids**	5/5	64% [28–89%]

(*) (17, 21, 29, 33, 35).

### Data Analysis From Regional Surveillance System Reports

Among the countries from the Region of the Americas, Brazil was one of the most severely affected, registering a total of 11,439,558 cumulative confirmed cases of COVID-19 in the general population and 277,102 deaths by March 13, 2021 (37). Data from the epidemiological surveillance system showed that in Brazil, among 14 epidemiological week reports in 2020 and 10 in 2021, a total of 813 cases of MIS-C and 51 deaths were recorded in children and adolescents aged 0–19 years, with a mortality rate of 6.3%.

Most of the cases belonged to patients under 10 years of age, with 41% in the age group 0–4 years, followed by 34% in the group 5–9 years; 56.7% were male.

When analyzing Pan American Health Organization (PAHO) epidemiological reports, between May 2020 to March 2021, Brazil notified 769 confirmed cases of MIS-C and 47 deaths, a relatively lower number than the cases registered in the country’s surveillance system for the same period analyzed (38, 39).

COVID-19 was reported in 19,433 cases in Chile during the period analyzed, 10.2% younger than 19 years old. Similarly, between the 15th weekly report in 2020 and the 12th in 2021, 174 MIS-C cases and 3 deaths were reported in children and adolescents under 19 years of age, with a mortality of 1.7%.

Regarding the temporal distribution of both events, the highest number of COVID-19 cases in children and adolescents was registered between the 23rd and 25th weekly epidemiological reports in 2020 and the 9th to 10th of 2021, while the highest number of MIS-C cases was reported a few weeks later, between the 25th and 28th weekly epidemiological reports in 2020 and the 10th of 2021, although in the latter period the absolute number of cases was lower. The mean age of MIS-C cases was 6 years (IQR 7–9 years) and more than half of the patients, 99 (57%), were male (40). Between May 2020 and March 10th, 2021, Chile reported to PAHO 157 cases of MIS-C and two deaths (39).

As of November 28, 2020, Ecuador recorded 190,909 confirmed cases of COVID-19 and 13,371 deaths (41). Until epidemiological week 48 in 2020, 128 suspected cases of MIS-C in children and adolescents were registered, with the highest number during epidemiological week 24–25. Distribution by age group, most of the cases belonged to the 5–9-year-old group (*n* = 45; 35.2%) and the 10–14-year-old group (*n* = 33; 25.8%), 61.7% were male (42, 43). Among the 127 reported cases, three required critical care. By October 8, 2020, Ecuador reported to PAHO 7 confirmed cases, 16 probable and 90 cases with suspected MIS-C, and no deaths.

On September 2, 2020, the Dominican Republic recorded 113,962 confirmed cases of COVID-19 in the general population and 2,128 deaths 0.41 in 40 weekly epidemiological reports during 2020, the same country reported 65 cases of MIS-C and two deaths in children and adolescents aged 0–15 years. Distribution by age group was like Brazil, with the 0–4 age group being the most affected followed by the 5–9 age group.

## Discussion

In our systematic review of MIS-C in LAC and analysis of data from regional surveillance systems performed we found significant heterogeneity. Most studies were case series. Countries included in our study were Argentina, Brazil, Chile, Colombia, Costa Rica, Mexico, Peru, and Venezuela. Health-Ministry data on MIS-C are very patchy and even the PAHO reports are deficient because limited information is available for Latin America and the Caribbean countries, related probably to the lack of mandatory notification of MIS-C in some countries.

Data from the epidemiological surveillance system show that during 2020, the highest number of MIS-C cases was observed around 3 weeks after the highest number of COVID-19 cases in children and adolescents in Chile. A 2–5-week time lag has been observed between the peak of COVID-19 cases within communities and the increase in MIS-C cases. This suggests that acquired immunity may play a role in its development.

Likewise, it has been observed that the number of MIS-C cases reported by PAHO is lower than that reported by the epidemiological reports of the countries within similar temporal parameters. Nevertheless, this may be related to under-registration of cases due to differences in epidemiological records. Also, the reporting of data from the central level may not reflect the behavior of the surveillance system at the subnational level since delays in reports could occur due to the registration of a large number of suspected cases of COVID-19. MIS-C case fatality rate for the same period was lower in Chile than in Brazil according to country surveillance reports. Chilean MIS-C cases and mortality reported by PAHO were slightly lower than those recorded by the country’s surveillance system for the same period. The pooled case fatality rate from 23 reports of 4% was slightly higher than the 1–2% overall reported rate from other regions (44).

Similar to other regions, in LAC countries more than half the patients were male (45). The median age of the included patients was 6.6 years, slightly lower than 8–9 years described in other populations. Apparently, the affected population in LAC is younger than in other regions, such as Europe and North America. It would be interesting to investigate possible causes, such as sociodemographic, ethnic, or other variables that could determine this difference (44, 46–49).

In general terms, the distribution of cases by sex was similar with a frequency of male sex between 51 and 58%. Gender was not related to severity of the clinical course; however, we observed that 73% of patients requiring PICU admission were male. In adults, male sex is a risk factor for severe COVID-19. Specifically, in a Latin-American study including data from patients with COVID-19 and MIS-C, girls had a lower frequency of hospitalization without differences in mortality among patients who developed MIS-C (44, 46–50).

In 59% of patients with MIS-C, close contact with a person with COVID-19 was described. This finding was more frequent than in other reviews reporting only 15–20% (44). The high percentage of household infections in Latin-America may be related to overcrowded housing conditions and other socio-demographic factors. In most epidemiological surveillance registries from Chile and other Latin American countries, most cases were confirmed by clinical or epidemiological criteria with a low frequency of concomitant positive SARS-CoV-2 tests.

Around one-third of the patients had one or more comorbidities. This has also been described in other reviews, with a frequency between 20 and 25%. The most observed comorbidities were obesity and/or overweight, chronic respiratory diseases, onco-hematological diseases, and neurological disorders (64). This finding confirms previous reports showing that MIS-C predominantly affects healthy individuals (44, 49, 51, 52). In most reviews, MIS-C diagnosis was made by serology in 60% to almost 90% of cases, while direct diagnostic methods such as PCR for SARS-CoV-2, were positive in swabs in less than of 40% of cases, similar to our findings. This would support the fact that MIS-C occurs as a post-infection phenomena (47, 48, 51, 53, 54).

Regarding the presenting signs and symptoms, fever was the most frequent followed by gastrointestinal manifestations, rash, and conjunctival injection. Neurological, respiratory, and cardiological manifestations were also reported. In most reports, fever and abdominal or gastrointestinal symptoms (abdominal pain, diarrhea, and vomiting) are described as the most frequent (46, 48, 53). On the other hand, the presenting symptoms of MIS-C may mimic an acute abdomen, including acute appendicitis, as shown in a multicenter Latin-American study, in which 34/1,010 (3.3%) patients with COVID-19 and MIS-C had an intraoperative diagnosis of appendicitis (55). Furthermore, a systematic review of abdominal pain in MIS-C found an incidence of acute abdomen of 19%, which was non-surgical in most cases. Patients who required surgery due to appendicitis or obstruction were 17/72 (23.6%) patients with acute abdomen (55, 56).

Mucocutaneous manifestations, such as rash were reported between 40 and 60%; conjunctivitis in 50%, similar to our study (52). Neurological involvement was less frequent, with headache, neck stiffness, and visual disturbances reported in 30% of cases (50). MIS-C patients generally have less respiratory symptoms than COVID-19 as reported in large series and reviews. In a systematic review, around 50% of the patients showed respiratory symptoms, including cough or dyspnea with radiological findings (51–54). A variable proportion of patients with MIS-C develop hypotension and shock because of acute myocardial dysfunction or hyperinflammatory state. In our study, hemodynamic involvement was less frequent compared to other reviews (48, 54, 57).

In the current review, 59% of MIS-C cases had Kawasaki-like syndrome. Kaushik et al. reported cardiac involvement in MIS-C patients with Kawasaki-like syndrome, of whom 20% had coronary artery dilatation/aneurysms and hypotension and 28% shock, like our findings. Despite some similar phenotypic characteristics between MIS-C and KD, there are other differential parameters, such as age less than 5 years in children with KD and the fact that approximately 7% of KD patients present with cardiovascular collapse (KDSS). In our study, 49% of the patients evolved to shock (58).

Inflammatory markers (CRP, D-dimer, and ferritin) were elevated and so was the incidence of lymphopenia. Feldstein et al. reported similar findings in patients with MIS-C compared to those with severe acute COVID-19 (8). In addition, in patients with MIS-C who required PICU admission we observed elevated inflammatory markers and lower lymphocyte and platelet counts compared to patients with MIS-C in general. These findings are consistent with other systematic reviews (53).

When comparing the overall analysis of patients included in our study with that of the subgroup of those with severe MIS-C, an increased male predominance (71% vs. 59%), a higher frequency of comorbidities, higher CRP levels, and lower lymphopenia counts are found. Different series of patients with severe MIS-C reported similar features (59, 60). Tripathi et al. reported a comorbidity rate (33%) similar to our study (61). Mortality was higher when compared to that of developed countries (7% vs. 3–4.7%), but lower than that observed in the Colombian MISCO study (9%) (61). It is likely that the delay in access to health care, diagnosis, and patient care explains, at least in part, this higher mortality and heterogeneity between countries in the region. In a report by Farias et al. a high frequency of pre-existing diseases and immunosuppression was found, which may have contributed to the high mortality rate.

IVIG and/or steroids are proposed as first-line treatment in patients with MIS-C, although to date there are no controlled studies comparing IVIG and corticosteroids alone or in combination. Some observational studies have found combination therapy to be beneficial. A French study including 111 patients with MIS-C found a lower rate of treatment failure in those who received combination therapy vs. IVIG as monotherapy (62). A US study in 518 MIS-C patients observed that combination therapy was associated with a lower risk of new or persistent cardiovascular dysfunction compared to IVIG alone. Both studies found a lower requirement for second-line therapy and hemodynamic support within 24–48 h after initial treatment (63). On the other hand, a large study carried out in multiple countries comparing three treatment regimens (combination therapy, IVIG, or corticosteroids alone) found no differences in morbidity and mortality. Nevertheless, the selection and outcome criteria were different in the three studies (45). In our study, most patients received IVIG, in combination with corticosteroids in more than half of the patients.

In agreement with a study by Pereira et al. (35) we observed an increased use of antibiotics, related to the main differential diagnoses of MIS-C, septic shock, and toxic shock syndrome. Antibiotic prescriptions among MIS-C patients may not have been appropriate in most cases and antimicrobial stewardship should be explored in MIS-C patients. Tocilizumab or siltuximab were used only in a small number of patients.

Other frequently used medications as part of treatment were aspirin and anticoagulants. The wide variety of treatments used may have been due to the lack of knowledge of MIS-C, especially in the first months after described in Europe. In addition, many treatments are unavailable in the region due to their high costs. Most importantly, there is still no clear consensus on the treatments to be administered beyond the recommended use of IVIG and corticosteroids.

PICU admissions were less frequent than in other reports (44, 48). One possible explanation is that at the moment that MIS-C started to be reported, no guidelines or alerts to detect and treat children with MIS-C were available. Use of resources in terms of length of hospital and PICU stay were like other reports (48).

Information available from LAC was found to be limited, probably related to the lack of mandatory notification of MIS-C locally. One of the key limitations of the study is that most of the data reported were from case series and were highly heterogeneous. To our knowledge this is the first systematic review with meta-analysis incorporating data from regional surveillance systems from LAC.

## Conclusion

The results of the present study provide important information to help understanding MIS -C in children and adolescents from LAC. Epidemiological information in the region was very limited. Optimization of case registration and surveillance is needed. Knowledge about this syndrome and the impact of the recent introduction of COVID-19 vaccination schedules in several Latin American countries is still insufficient.

## Author Contributions

SRu and AB conceived the study. CV, MR, AF, NV, MB, SRo, and RU-G collected and analyzed the studies included. SRu, CV, MR, AF, NV, MB, SRo, RU-G, and AB drafted the manuscript. All authors contributed and approved the final manuscript.

## Conflict of Interest

The authors declare that the research was conducted in the absence of any commercial or financial relationships that could be construed as a potential conflict of interest.

## Publisher’s Note

All claims expressed in this article are solely those of the authors and do not necessarily represent those of their affiliated organizations, or those of the publisher, the editors and the reviewers. Any product that may be evaluated in this article, or claim that may be made by its manufacturer, is not guaranteed or endorsed by the publisher.
