# Supplementary material for: Multisystem Inflammatory Syndrome Temporally Related to COVID-19 in Children From Latin America and the Caribbean Region: A Systematic Review With a Meta-Analysis of Data From Regional Surveillance Systems
Source: Front Pediatr. 2022 Apr 25;10:881765. doi: 10.3389/fped.2022.881765 (PMC9082071; doi:10.3389/fped.2022.881765)
Supplement: Supplementary file 1 [file Data_Sheet_1.pdf]

Supplemental Material

Table A1

Section and Topic	Item #	Checklist item	Location where item is reported (page)
**TITLE**			
Title	1	Identify the report as a systematic review.	1
**ABSTRACT**			
Abstract	2	See the PRISMA 2020 for Abstracts checklist.	4
**INTRODUCTION**			
Rationale	3	Describe the rationale for the review in the context of existing knowledge.	4
Objectives	4	Provide an explicit statement of the objective(s) or question(s) the review addresses.	4
**METHODS**			
Eligibility criteria	5	Specify the inclusion and exclusion criteria for the review and how studies were grouped for the syntheses.	4
Information sources	6	Specify all databases, registers, websites, organisations, reference lists and other sources searched or consulted to identify studies. Specify the date when each source was last searched or consulted.	4
Search strategy	7	Present the full search strategies for all databases, registers and websites, including any filters and limits used.	4
Selection process	8	Specify the methods used to decide whether a study met the inclusion criteria of the review, including how many reviewers screened each record and each report retrieved, whether they worked independently, and if applicable, details of automation tools used in the process.	4- 5 (fig 1)
Data collection process	9	Specify the methods used to collect data from reports, including how many reviewers collected data from each report, whether they worked independently, any processes for obtaining or confirming data from	5- 6

		study investigators, and if applicable, details of automation tools used in the process.	
Data items	10a	List and define all outcomes for which data were sought. Specify whether all results that were compatible with each outcome domain in each study were sought (e.g. for all measures, time points, analyses), and if not, the methods used to decide which results to collect.	4- 5
	10b	List and define all other variables for which data were sought (e.g. participant and intervention characteristics, funding sources). Describe any assumptions made about any missing or unclear information.	NO
Study risk of bias assessment	11	Specify the methods used to assess risk of bias in the included studies, including details of the tool(s) used, how many reviewers assessed each study and whether they worked independently, and if applicable, details of automation tools used in the process.	5
Effect measures	12	Specify for each outcome the effect measure(s) (e.g. risk ratio, mean difference) used in the synthesis or presentation of results.	5
Synthesis methods	13a	Describe the processes used to decide which studies were eligible for each synthesis (e.g. tabulating the study intervention characteristics and comparing against the planned groups for each synthesis (item #5)).	4
	13b	Describe any methods required to prepare the data for presentation or synthesis, such as handling of missing summary statistics, or data conversions.	NO
	13c	Describe any methods used to tabulate or visually display results of individual studies and syntheses.	4
	13d	Describe any methods used to synthesize results and provide a rationale for the choice(s). If meta-analysis was performed, describe the model(s), method(s) to identify the presence and extent of statistical heterogeneity, and software package(s) used.	12
	13e	Describe any methods used to explore possible causes of heterogeneity among study results (e.g. subgroup analysis, meta-regression).	4
	13f	Describe any sensitivity analyses conducted to assess robustness of the synthesized results.	NO

Reporting bias assessment	14	Describe any methods used to assess risk of bias due to missing results in a synthesis (arising from reporting biases).	12
Certainty assessment	15	Describe any methods used to assess certainty (or confidence) in the body of evidence for an outcome.	NO
**RESULTS**			
Study selection	16a	Describe the results of the search and selection process, from the number of records identified in the search to the number of studies included in the review, ideally using a flow diagram.	Figure 1
	16b	Cite studies that might appear to meet the inclusion criteria, but which were excluded, and explain why they were excluded.	Table A6, page 47
Study characteristics	17	Cite each included study and present its characteristics.	Table 1
Risk of bias in studies	18	Present assessments of risk of bias for each included study.	12
Results of individual studies	19	For all outcomes, present, for each study: (a) summary statistics for each group (where appropriate) and (b) an effect estimate and its precision (e.g. confidence/credible interval), ideally using structured tables or plots.	Table 2- 6
Results of syntheses	20a	For each synthesis, briefly summarize the characteristics and risk of bias among contributing studies.	NO
	20b	Present results of all statistical syntheses conducted. If meta-analysis was done, present for each the summary estimate and its precision (e.g. confidence/credible interval) and measures of statistical heterogeneity. If comparing groups, describe the direction of the effect.	Table 2-4, plots
	20c	Present results of all investigations of possible causes of heterogeneity among study results.	Plots
	20d	Present results of all sensitivity analyses conducted to assess the robustness of the synthesized results.	NO
Reporting biases	21	Present assessments of risk of bias due to missing results (arising from reporting biases) for each synthesis assessed.	

Certainty of evidence	22	Present assessments of certainty (or confidence) in the body of evidence for each outcome assessed.	NO
**DISCUSSION**			
Discussion	23a	Provide a general interpretation of the results in the context of other evidence.	27
	23b	Discuss any limitations of the evidence included in the review.	26
	23c	Discuss any limitations of the review processes used.	22
	23d	Discuss implications of the results for practice, policy, and future research.	27
**OTHER INFORMATION**			
Registration and protocol	24a	Provide registration information for the review, including register name and registration number, or state that the review was not registered.	2
	24b	Indicate where the review protocol can be accessed, or state that a protocol was not prepared.	NO
	24c	Describe and explain any amendments to information provided at registration or in the protocol.	NO
Support	25	Describe sources of financial or non-financial support for the review, and the role of the funders or sponsors in the review.	24
Competing interests	26	Declare any competing interests of review authors.	24
Availability of data, code and other materials	27	Report which of the following are publicly available and where they can be found: template data collection forms; data extracted from included studies; data used for all analyses; analytic code; any other materials used in the review.	NO

From: Page MJ, McKenzie JE, Bossuyt PM, Boutron I, Hoffmann TC, Mulrow CD, et al. The PRISMA 2020 statement: an updated guideline for reporting systematic reviews. BMJ 2021;372:n71. doi: 10.1136/bmj.n71

For more information, visit: http://www.prisma-statement.org/

Table A2: risk of bias of cohort and cross-sectional studies

Study ID	Study design	1	2	3	4	5	6	7	8	9	10	11	12	13	14	Global Quality
Coronado Muñoz et al. 2021[12]	Cohort	Yes	Yes	NR	Yes	NA	Yes	Yes	NA	Yes	No	Yes	No	NA	No	Fair
Seery et al. 2021[13]	Cohort	Yes	Yes	NR	Yes	Yes	Yes	Yes	Yes	Yes	No	Yes	No	NA	No	Good
Torres et al. 2020[15]	Cohort	Yes	Yes	NR	Yes	NA	Yes	Yes	NA	Yes	No	Yes	No	NA	No	Fair
Lima-Setta et al. 2020[19]	Cohort	Yes	Yes	NR	Yes	NA	Yes	Yes	NA	Yes	No	Yes	No	NA	No	Fair
Antúnez-Montes et al. 2021[29]	Cohort	Yes	Yes	NR	Yes	NA	Yes	Yes	NA	Yes	No	Yes	No	NA	No	Fair
Prata-Barbosa et al. 2020[31]	Cohort	Yes	Yes	NR	Yes	NA	Yes	Yes	NA	Yes	No	Yes	No	NA	Yes	Good
Yock-Corrales et al. 2021[32]	Cohort	Yes	Yes	NR	Yes	No	Yes	Yes	NA	Yes	No	Yes	No	NA	Yes	Good
Dominguez Rojas	Cross section	Yes	Yes	NR	Yes	NA	Yes	Yes	NA	Yes	No	Yes	No	NA	No	Fair

Badue Pereira	Cross section	Yes	Yes	NR	Yes	NA	Yes	Yes	NA	Yes	No	No	No	NA	No	Fair

Was the research question or objective in this paper clearly stated?
Was the study population clearly specified and defined
Was the participation rate of eligible persons at least 50%?
Were all the subjects selected or recruited from the same or similar populations (including the same time period)? Were inclusion and exclusion criteria for being in the study prespecified and applied uniformly to all participants?
Was a sample size justification, power description, or variance and effect estimates provided?
For the analyses in this paper, were the exposure(s) of interest measured prior to the outcome(s) being measured?
Was the timeframe sufficient so that one could reasonably expect to see an association between exposure and outcome if it existed?
For exposures that can vary in amount or level, did the study examine different levels of the exposure as related to the outcome (e.g., categories of exposure, or exposure measured as continuous variable)?
Were the exposure measures (independent variables) clearly defined, valid, reliable, and implemented consistently across all study participants?
Was the exposure(s) assessed more than once over time?
Were the outcome measures (dependent variables) clearly defined, valid, reliable, and implemented consistently across all study participants?
Were the outcome assessors blinded to the exposure status of participants?
Was loss to follow-up after baseline 20% or less?
Were key potential confounding variables measured and adjusted statistically for their impact on the relationship between exposure(s) and outcome(s)?
*CD, cannot determine; NA, not applicable; NR, not reported

Table A3: Risk of bias of case series studies

Study ID	Study design	1	2	3	4	5	6	7	8	9	Global quality
Brenes Chacón	Case series	No	No	NR	NR	NA	No	NR	No	No	Poor
Gutiérrez Hidalgo	Case series	No	No	NR	CD	NA	No	NR	No	No	Poor
Luna Muñoz	Case series	Yes	Yes	CD	Yes	NA	Yes	Yes	No	No	Fair
De Farías	Case series	Yes	Yes	NR	Yes	NA	Yes	NR	No	Yes	Fair
Coll- Vela	Case series	Yes	Yes	NR	Yes	NA	Yes	Yes	NR	Yes	Fair
Duarte Neto	Case series	No	No	NR	Yes	NA	Yes	NA	No	Yes	Poor
Sandoval	Case series	Yes	Yes	NR	Yes	NA	Yes	CD	No	Yes	Fair
Coila Paricahua	Case series	Yes	No	NR	Yes	NA	No	Yes	No	No	Poor
Fontes	Case series	Yes	No	NR	Yes	NA	Yes	CD	Yes	Yes	Fair
Del Aguila	Case series	Yes	CD	NR	Yes	NA	No	CD	No	Yes	Fair
Alvarez	Case series	Yes	Yes	CD	Yes	NA	Yes	Yes	Yes	Yes	Good
Niño Taravilla	Case series	Yes	Yes	CD	Yes	NA	No	Yes	Yes	Yes	Fair
Ivankovich Escoto	Case series	Yes	No	NR	Yes	NA	No	No	No	No	Poor

Was the study question or objective clearly stated?

Was the study population clearly and fully described, including a case definition?
Were the cases consecutive?
Were the subjects comparable?
Was the intervention clearly described?
Were the outcome measures clearly defined, valid, reliable, and implemented consistently across all study participants?
Was the length of follow-up adequate?
Were the statistical methods well-described?
Were the results well-described?
*CD, cannot determine; NA, not applicable; NR, not reported

Table A4: Risk of bias case control study

Study ID	Study design	1	2	3	4	5	6	7	8	9	10	11	12	Global quality
Rosanova	Case control	Yes	Yes	No	Yes	Yes	Yes	No	Yes	No	Yes	No	Yes	Fair

Was the research question or objective in this paper clearly stated and appropriate?
Was the study population clearly specified and defined?
Did the authors include a sample size justification?
Were controls selected or recruited from the same or similar population that gave rise to the cases (including the same timeframe)?

Were the definitions, inclusion and exclusion criteria, algorithms or processes used to identify or select cases and controls valid, reliable, and implemented consistently across all study participants?
Were the cases clearly defined and differentiated from controls?
If less than 100 percent of eligible cases and/or controls were selected for the study, were the cases and/or controls randomly selected from those eligible?
Was there use of concurrent controls?
Were the investigators able to confirm that the exposure/risk occurred prior to the development of the condition or event that defined a participant as a case?
Were the measures of exposure/risk clearly defined, valid, reliable, and implemented consistently (including the same time period) across all study participants?
Were the assessors of exposure/risk blinded to the case or control status of participants?
Were key potential confounding variables measured and adjusted statistically in the analyses? If matching was used, did the investigators account for matching during study analysis?

*CD, cannot determine; NA, not applicable; NR, not reported

Table A5: studies excluded during full-text screening phase

**Author**	**n**	**Study design**	**Reason for exclusion**
Caro Domínguez[63]	37	Case series	No specific information from LAC
Lindan[64]	38	Case series	No MIS-C in children from LAC
Kehar[65]	91	Cohort	No specific information from LAC
Bautista Rodríguez[66]	183	Case series	No specific information from LAC

Table A6: Detailed characteristics of SARS-CoV-2 RT-PCR, serology test results, and Kawasaki and shock presentations in MIS-C patients

Author	Number	Country	PCR +	Serology +	Close contact of COVID-19	Kawasaki disease	Shock
**Seery et al. – 2021**	21	Argentina	10 (47.6%)	19 (90.5%)	16 (76.2%)	NR	5 (23.8%)
**Coila-Paricahua et al. – 2021**	13	Peru	0	13 (100%)	NR	NR	NR
**Torres J. et al – 2020**	27	Chile	14 (51.85%)	10 (37%)	9 (33.3%)	4 (14.8%)	16 (59%)
**Badue Pereira et al. – 2021**	6	Brazil	5 (83.3%)	1 (16.67%)	NR	3 (50%)	5 (83.3%)
**Rosanova et al. – 2021**	25	Argentina	15 (60%)	24 (96%)	1 (4%)	NR	8 (32%)
**Coll-Vela – 2021**	8	Peru	1 (12.5%)	8 (100%)	7 (87.5%)	7 (87.5%)	1 (12.5%)
**Coronado Muñoz – 2021**	21	Peru	NR	NR	NR	13 (61.9%)	NR
**Sandoval – 2021**	17	Chile	8 (47%)	10 (58.8%)	NR	13 (48%)	15 (88.4%)
**Yock Corrales – 2021**	69	Argentina, Colombia, Costa Rica, Mexico, Peru	16 (23.2%)	49 (71%)	39 (56.5%)	NR	NR
**Fontes – 2021**	42	Brazil	5 (11.9%)	6 (14.3%)	8 (19%)	NR	NR
**Lima-Setta – 2020**	56	Brazil	14 (25%)	19 (33.9%)	NR	42 (75%)	10 (17.8%)
**Aguila – 2021**	37	Peru	3 (8.1%)	32 (86.5%)	NR	18 (48.65%)	8 (21.6%)
**Álvarez – 2020**	23	Chile	11 (47.8%)	13 (56.5%)	12 (52.2%)	18 (78.26%)	9 (39.13%)
**Niño-Taravilla – 2021**	26	Chile, Colombia, Peru, Others	7 (26.9%)	15 (57.7%)	10 (38.4%)	16 (61.54%)	24 (92.3%)

**Ivankovich-Escoto – 2021**	11	Costa Rica	5 (45.45%)	10 (90.9%)	8 (72.7%)	NR	NR
**Brenes-Chacón – 2021**	26	Costa Rica	16 (61.5%)	18 (69.2%)	NR	NR	15 (57.7%)
**Gutiérrez-Hidalgo – 2021**	4	Costa Rica	NR	NR	NR	NR	4 (100%)
**Luna-Muñoz – 2021**	10	Peru	NR	10 (100%)	8 (80%)	NR	5 (50%)
**de Farias – 2021**	11	Brazil	2 (18.2%)	9 (81.8%)	10 (91%)	9 (82%)	8 (72.7%)
**Domínguez Rojas – 2021**	31	Peru	1 (3,2%)	29 (93.55%)	13 (42%)	12 (38.7%)	9 (29%)
**Duarte-Neto – 2021**	3	Brazil	1 (33.3%)	1 (33,33%)	2 (66,67%)	NR	3 (100%)
**Prata Barbosa – 2020**	10	Brazil	5 (50%)	6 (60%)	10 (100%)	6 (60%)	NR
**Antunez-Montes – 2021**	95	NR	NR	NR	NR	NR	NR

abbreviation: NR = Not reported

Figure A1: Forest plots of laboratory test results in patients with MIS-C: (A) Platelets, (B) Lymphocytes, (C) CRP, (D) Troponin, (E) Ddimer, (F) Albumin.

(A) Platelets

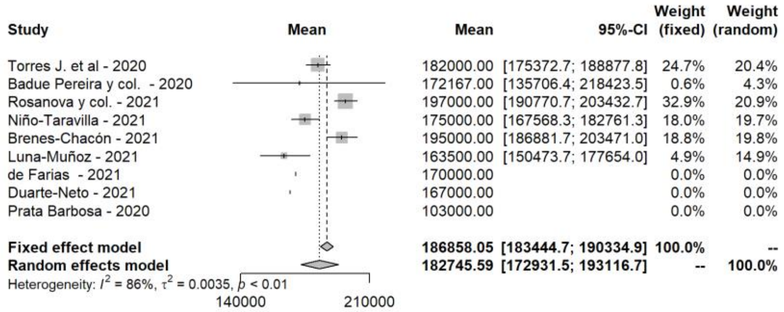

(B)Lymphocytes

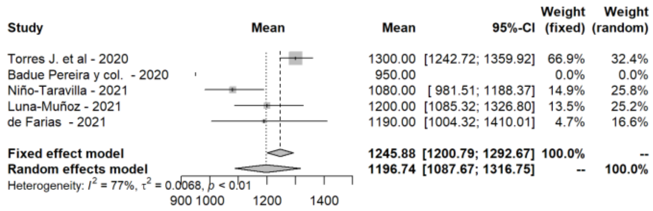

(C)CRP

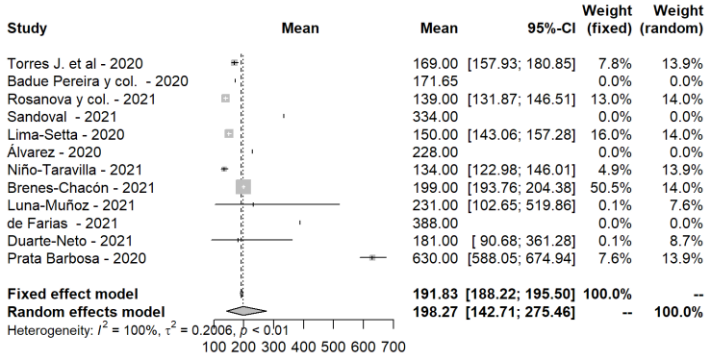

(D)Troponin

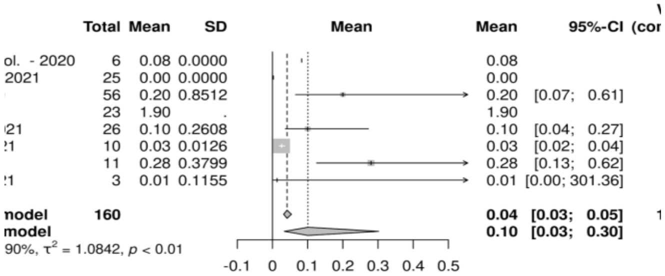

(E)DDimer

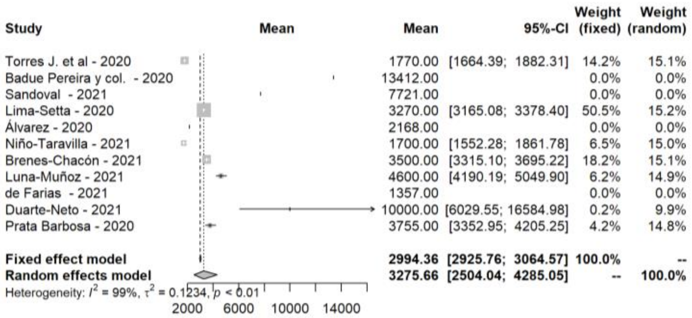

(F) Albumina

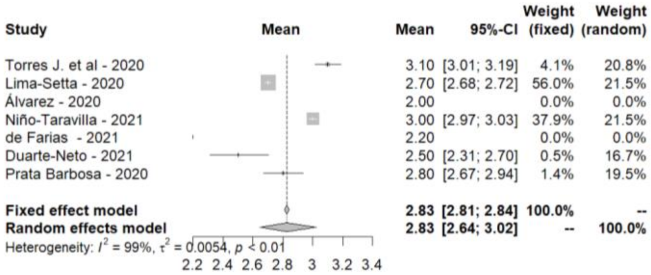

Table A7: Cardiac involvement in patients with and without Kawasaki disease

Author	Number	Kawasaki disease	Abnormal echocardiogram	Pericarditis	Myocarditis	Ventricular dysfunction	Coronary aneurysm/ dilation/abnormality	Valvular dysfunction	Abnormal ECG	Raised proBNP	Raised Troponin
Torres J. et al - 2020	27	4 (14.81%)	12 (44.4%)	12 (44.4%)	NR	4 (14.81%)	5 (18.5%)	NR	NR	NR	NR
Badue Pereira et al. - 2021	6	3 (50%)	6 (100%)	5 (83.33%)	1 (16.67%)	3 (50%)	3 (50%)	NR	NR	NR	NR
Coll-Vela - 2021	8	7 (87.5%)	2 (25%)	2 (25%)	2 (25%)	2 (25%)	NR	1 (12,5%)	1 (12.5%)	NR	NR
Coronado Muñoz - 2021	21	13 (61%)	NR	1 (5%)	NR	NR	NR	NR	NR	NR	NR
Sandoval - 2021	17	13 (76%)	NR	NR	NR	NR	NR	NR	NR	NR	NR
Lima-Setta - 2020	56	42 (75%)	34 (61%)	17 (30.36%)	NR	9 (16.1%)	9 (16%)	NR	NR	27 (48%)	24 (42%)
Aguila - 2021	37	18 (48.6%)	NR	NR	NR	NR	5 (13.5%)	NR	NR	4 (10.8%)	NR
Álvarez - 2020	23	18 (78.2%)	NR	13 (56.5%)	8 (34.78%)	NR	3 (13%)	2 (8.7%)	6 (26.1%)	4 (17%)	10 (43.5%)
Niño-Taravilla - 2021	26	16 (61.4%)	18 (69%)	3 (11.54%)	12 (46.1%)	9 (34.6%)	5 (19.2%)	NR	NR	NR	NR
de Farias - 2021	11	9 (82%)	7 (63.64%)	6 (54.5%)	NR	NR	7 (64%)	4 (36%)	5 (45.45%)	NR	7 (64%)
Domínguez Rojas - 2021	31	12 (38.71%)	8 (25.81%)	NR	NR	8 (25.81%)	1 (3.23%)	NR	NR	NR	NR

Prata Barbosa - 2020	10	6 (60%)	NR	NR	NR	NR	NR	NR	NR	6 (60%)	NR
**Without Kawasaki**											
Seery et al. - 2021	21	0	NR	NR	NR	NR	NR	NR	NR	NR	NR
Coila-Paricahua et al. - 2021	13	NR	NR	NR	NR	NR	NR	NR	NR	NR	NR
Rosanova et al. - 2021	25	NR	8 (32%)	1 (4%)	3 (12%)	3 (12%)	1 (4%)	NR	NR	NR	NR
Yock Corrales - 2021	69	NR	NR	NR	NR	NR	NR	NR	NR	NR	NR
Fontes - 2021	42	NR	NR	NR	NR	NR	NR	NR	NR	NR	NR
Ivankovich-Escoto - 2021	11	NR	NR	NR	NR	NR	NR	NR	NR	NR	NR
Brenes-Chacón - 2021	26	NR	17 (65.38%)	5 (19.23%)	1 (3,85%)	NR	4 (15.38%)	NR	NR	NR	NR
Gutiérrez-Hidalgo - 2021	4	NR	NR	NR	NR	NR	NR	NR	NR	NR	NR
Luna-Muñoz - 2021	10	NR	NR	NR	NR	2 (20%)	2 (20%)	NR	NR	5 (50%)	3 (30%)
Duarte-Neto - 2021	3	NR	3 (100%)	1 (33.33%)	2 (66.67%)	2 (66.67%)	1 (33.33%)	NR	NR	NR	1 (33.33%)

Antunez-Montes - 2021	95	NR	NR	4 (4.21%)	2 (2.11%)	NR	5 (5.26%)	NR	NR	NR	NR

abbreviation: NR = Not reported

Table A8: MIS-C Patients admitted to the PICU and use of resources

**Author**	**Number**	**PICU (LOS) days (Mean (SD)**	**Hospitalization days LOS (Mean (SD))**
Coila-Paricahua et al.-2021	13	6 (0.7)	NR
Torres J. et al - 2020	27	NR	3 (5.2)
Badue Pereira et al. - 2021	6	1	NR
Rosanova et al. - 2021	25	NR	10 (2.2)
Sandoval - 2021	17	9	13
Fontes - 2021	42	1	NR
Lima-Setta - 2020	56	56 (4.8)	NR
Álvarez - 2020	23	NR	23
Niño-Taravilla - 2021	26	NR	19 (7)
Brenes-Chacón - 2021	26	NR	8.7(8.7)
Gutiérrez-Hidalgo - 2021	4	NR	11.5
Luna-Muñoz - 2021	10	NR	10.4 (3)
de Farias - 2021	11	11 (3)	17 (11.9)
Domínguez Rojas - 2021	31	NR	8
Prata Barbosa - 2020	10	10 (3.5)	0

abbreviation: NR = Not reported

Table A9 . Treatments used in MIS-C patients in Latin America and the Caribbean

Authors	Number	Oxygen	Intravenous immunoglobulin	Intravenous immunoglobulin (2nd dose)	Steroids	Aspirin	Anticoagulation	Antibiotics
Seery et al. - 2021	21	1 (4.8%)	20 (95.2%)	NR	20 (95%)	15 (71%)	14 (66.7%)	2 (9.5%)
Torres J. et al - 2020	27	13 (48%)	19 (70.4%)	NR	17 (63%)	17 (63%)	18 (66.,7%)	24 (88.9%)
Badue Pereira et al -2021	6	6	4 (66.7%)	NR	2 (33.3%)	3 (50%)	2 (33.3%)	6
Rosanova et al. - 2021	25	NR	25	NR	15 (60%)	NR	NR	NR
Coll-Vela - 2021	8	1 (12%)	8	2 (25%)	8	8	6 (75%)	8
Coronado Muñoz - 2021	21	NR	15 (71.4%)	NR	8 (38.1%)	15 (71%)	8 (38.1%)	NR
Sandoval - 2021	17	NR	7 (41.2%)	NR	7 (41.2%)	NR	8 (47.1%)	NR
Yock Corrales - 2021	69	29 (42%)	60 (87%)	NR	43 (62%)	NR	NR	56 (81.2%)
Lima-Setta - 2020	56	14 (25%)	50 (89.3%)	NR	31 (55%)	25 (44%)	29 (51.8%)	33 (58.9%)
Aguila - 2021	37	NR	36 (97.3%)	10 (27%)	21 (57%)	33 (89%)	4 (10.8%)	27 (73%)
Álvarez - 2020	23	NR	20 (87%)	3 (13%)	17 (74%)	22 (96%)	17 (73.9%)	NR
Niño-Taravilla - 2021	26	NR	22 (84.6%)	2 (7.7%)	23 (88%)	19 (73%)	19 (73.1%)	NR
Brenes-Chacón-2021	26	8 (30.8%)	26	2 (7.7%)	11 (42%)	NR	NR	NR
Luna-Muñoz - 2021	10	1 (10%)	10	NR	7 (70%)	7 (70%)	1 (10%)	8 (80%)
de Farias - 2021	11	NR	10 (90.9%)	NR	11	9 (81,8%)	9 (81,8%)	NR
Domínguez Rojas - 2021	31	15 (48%)	14 (45%)	NR	10 (32%)	14 (45%)	2 (6.5%)	27 (87.1%)
Duarte-Neto - 2021	3	NR	2 (66.7%)	1 (33.3%)	1 (33.3%)	NR	NR	NR
Prata Barbosa - 2020	10	4 (40%)	NR	NR	2 (20%)	NR	NR	8 (80%)
Antunez-Montes - 2021	95	25 (26%)	38 (40%)	NR	27 (28%)	NR	NR	NR

abbreviation: NR = Not reported

Seven patients received Tocilizumab or Siltuximab

Table A10: Clinical and laboratory features and outcome in MIS-C patients admitted to PICU

**Variables**	**Torres et al**	**Coll-vela et al**	**Badue Pereira et al**	**Lima-Setta**	**de Farias**	**Prata Barbosa**
Total patients	27	8	6	56	11	10
PICU requirement	16 (59.26%)	1 (12.5%)	6	56	11	10
Country	Chile	Peru	Brazil	Brazil	Brazil	Brazil
Age: Mean (SD)	6.5 (10.3)	9	7.8	6.2 (5.8)	4.3 (8.15)	5.2 (5.1)
Male	9 (56.25%)	1	5 (83.33%)	39 (69.64%)	9 (81.8%)	8 (80%)
One or more comorbidities	4	0	9	10	9	2
Fever	16	1	6	56	11	8 (80%)
Mucocutaneous manifestations	23	3	NR	96	NR	NR
Respiratory manifestations	4	0	20	49	4	15
Gastrointestinal manifestations	28	3	NR	97	NR	15
Hypotension	NR	NR	3 (50%)	17 (30.36%)	NR	2 (20%)
Kawasaki Disease	NR	NR	1 (16,67%)	42 (75%)	5 (45,45%)	6 (60%)
Shock	NR	NR	5 (83.3%)	10 (17.86%)	8 (72.7%)	NR
Lymphocytes: Mean (SD)	900 (800) cell/mm3	550 cell/mm3	950 cell/mm3	NR	1190 (1133) cell/mm3	NR
Platelets: Mean (SD)	150000 (105925) cell/mm3	122000 cell/mm3	172167 (125423) cell/mm3	NR	170000 (77777) cell/mm3	103000 (73148) cell/mm3
PCR: Mean (SD)	18 (12.5) mg/dl	16.7 mg/dl	17.1 mg/dl	15 (20.3) mg/dl	38.8 mg/dl	63 (22) mg/dl
Ferritin: Mean (SD)	431 (319) ng/ml	495 ng/ml	3660 ng/ml	464 (493) ng/ml	619 (469) ng/ml	228 (906) ng/ml
Procalcitonin: Mean (SD)	NR	NR	NR	1 (1.5) ng/ml	NR	15.7 (11) ng/ml
Troponin: Mean (SD)	NR	NR	NR	0.2 (6.37) ng/ml	0.28 (1.2) ng/ml	0 (0.03) ng/ml
D Dimer: Mean (SD)	2350 ng/ml	3680 ng/ml	1341 ng/ml	3270 (3047) ng/ml	1357 ng/ml	3755 (2169) ng/ml

ProNRBNP: Mean (SD)	NR	NR	NR	5818 (8996) pg/ml	NR	5829 pg/ml
Albumin: Mean (SD)	2.9 (0.44) g/dL	2.8 g/dL	NR	2.7 (0.59) g/dL	2.2 g/dL	2.8 (0.59) g/dL
Thorax X-Ray abnormal	NR	1	5 (83.33%)	25 (44.64%)	NR	7 (70%)
Thorax CT abnormal	NR	NR	3 (50%)	11 (19.64%)	7 (63.64%)	1 (10%)
Oxygen	12 (75%)	NR	NR	14 (25%)	NR	4 (40%)
Non-invasive ventilation	NR	0	NR	3 (5.36%)	4 (36.36%)	1 (10%)
Mechanical ventilation	12 (75%)	NR	5 (83.33%)	6 (10.71%)	3 (27.27%)	1 (10%)
Inotropes	12 (75%)	NR	5 (83.33%)	NR	7 (63.64%)	NR
IVIG*	14 (87.5%)	NR	4 (66.67%)	50 (89.29%)	10 (90.91%)	NR
Steroids	13 (81.25%)	0	2 (33.33%)	31 (55.36%)	11	2 (20%)
Aspirin	13 (81.25%)	NR	3 (50%)	25 (44.64%)	9 (81.82%)	NR
Anticoagulation	12 (75%)	NR	2 (33.33%)	29 (51.79%)	9 (81.82%)	NR
Antibiotics	NR	NR	NR	33 (58.93%)	NR	8 (80%)
Antifungals	NR	NR	NR	4 (7.14%)	NR	1 (10%)
PICU LOS (Mean (SD)	NR	NR	NR	6 (4.81)	5 (2.96)	5.5 (3.48)
Hospitalization days LOS (Mean (SD)	12	14	NR	NR	17 (11.85)	NR
Death	0	0	4 (66.67%)	1 (1.79%)	2 (18.18%)	0

* one patient (Coll-Vella) required a second dose of IVIG. abbreviation: NR = Not reported.

Search strategies

PubMed 30/6/2021

Search	Query	Results
#32	#29 AND #30 Filters: from 2021/5/18 - 3000/12/12	18
#31	#29 AND #30	107
#30	(Americas[MeSH Terms:noexp] OR America*[tiab] OR Latin America[Mesh] OR Latin America*[tiab] OR Latinamerica*[tiab] OR Latinoamerica*[tiab] OR Latin*[tiab] OR Hispanic Americans[Mesh] OR Hispanic America*[tiab] OR Hispanoamerica*[tiab] OR Iberoamerica*[tiab] OR Ibero Americ*[tiab] OR Panamerican*[tiab] OR Central America[Mesh] OR Central America*[tiab] OR Centroamerica*[tiab] OR Mesoamerica*[tiab] OR Meso America*[tiab] OR Middle America*[tiab] OR South America[Mesh] OR South America*[tiab] OR Southamerica*[tiab] OR Sudamerica*[tiab] OR "America del sur"[tiab] OR Caribbean Region[Mesh] OR Caribbean[tiab] OR Caribe*[tiab] OR West Indies[Mesh] OR West Indi*[tiab] OR Antill*[tiab] OR Indians, South American[Mesh] OR Indians, Central American[Mesh] OR Amerindian*[tiab] OR Indians[tiab] OR American Indian*[tiab] OR Native America*[tiab] OR Patagoni*[tiab] OR Andes[tiab] OR Andean*[tiab] OR Amazon*[tiab] OR Argentin*[ad] OR Argentin*[tiab] OR Argentina[pl] OR Bolivia*[ad] OR Bolivia*[tiab] OR Bolivia[pl] OR Brazil*[ad] OR Brasil*[ad] OR Brazil*[tiab] OR Brasil*[tiab] OR Brazil[pl] OR Colombia*[ad] OR Colombia*[tiab] OR Colombia[pl] OR Chile*[ad] OR Chile*[tiab] OR Chile[pl] OR Ecuador*[ad] OR Ecuator*[ad] OR Ecuador*[tiab] OR Ecuador[pl] OR Guiana*[ad] OR Guiana*[tiab] OR French Guiana[pl] OR Guyan*[ad] OR Guyan*[tiab] OR Guyana[pl] OR Paraguay*[ad] OR Paraguay*[tiab] OR Paraguay[pl] OR Peru*[ad] OR Peru*[tiab] OR Peru[pl] OR Surinam*[ad] OR Surinam*[tiab] OR Suriname[pl] OR Uruguay*[ad] OR Uruguay*[tiab] OR Uruguay[pl] OR Venez*[ad] OR Venez*[tiab] OR Venezuela[pl] OR Belize*[ad] OR Belize*[tiab] OR Belize[pl] OR Costa Ric*[ad] OR Costarric*[ad] OR Costaric*[ad] OR Costa Ric*[tiab] OR Costarric*[tiab] OR Costaric*[tiab] OR Costa Rica[pl]	1,590,617

Search	Query	Results
	OR Salvador*[ad] OR Salvador*[tiab] OR El Salvador[pl] OR Guatemal*[ad] OR Guatemal*[tiab] OR Guatemala[pl] OR Hondur*[ad] OR Hondur*[tiab] OR Honduras[pl] OR Nicaragu*[ad] OR Nicaragu*[tiab] OR Nicaragua[pl] OR Panam*[ad] OR Panam*[tiab] OR Panama[pl] OR Mexico[Mesh] OR Mexic*[ad] OR Mexic*[tiab] OR Mejic*[tiab] OR Mexico[pl] OR Cuba*[ad] OR Cuba*[tiab] OR Cuba[pl] OR Dominic*[ad] OR Dominic*[tiab] OR Dominican Republic[pl] OR Haiti*[ad] OR Haiti*[tiab] OR Haiti[pl] OR Jamaic*[ad] OR Jamaic*[tiab] OR Jamaica[pl] OR Puerto Rico[Mesh] OR Puerto Ric*[tiab] OR Puertorric*[tiab] OR Puertoric*[tiab])	
#29	#1 OR #28	942
#28	#21 AND #27	866
#27	#22 OR #23 OR #24 OR #25 OR #26	2,808
#26	PIMS[tiab]	1,346
#25	MIS-C[tiab]	492
#24	MISC[tiab]	592
#23	Multi-System Inflammatory[tiab]	104
#22	Multisystem Inflammatory[tiab]	1,229
#21	#2 OR #3 OR #4 OR #5 OR #6 OR #7 OR #8 OR #9 OR #10 OR #11 OR #12 OR #13 OR #14 OR #15 OR #16 OR #17 OR #18 OR #19 OR #20	165,307
#20	N-Cov[tiab]	18
#19	CV-19*[tiab]	74
#18	CV19*[tiab]	23
#17	HCov*[tiab]	1,076
#16	Corono Virus[tiab]	1
#15	Virus Corona[tiab]	2,776

Search	Query	Results
#14	Coronovir*[tiab]	57
#13	Coronavir*[tiab]	69,583
#12	(Pneumonia[tiab] AND Wuhan[tiab] AND 2019[tiab])	1,136
#11	SARSCoV2[tiab]	41,196
#10	SARS-CoV2[tiab]	2,077
#9	SARS-CoV-2[tiab]	48,009
#8	2019-nCoV[tiab]	1,672
#7	COVID19*[tiab]	125,014
#6	COVID-19[tiab]	130,393
#5	Corona Virus[tiab]	2,107
#4	COVID-19[Mesh]	88,298
#3	SARS-CoV-2[Mesh]	68,507
#2	Coronavirus[Mesh]	81,022
#1	Pediatric Multisystem Inflammatory Disease, COVID-19 Related[Supplementary Concept]	452

Cochrane Library 30/6/2021

ID	Search	Hits
#1	MeSH descriptor: [Coronavirus] explode all trees	356
#2	MeSH descriptor: [SARS-CoV-2] explode all trees	343
#3	MeSH descriptor: [COVID-19] explode all trees	466
#4	(Coron* NEAR/1 Virus):ti,ab,kw	239
#5	COVID-19:ti,ab,kw	5915
#6	COVID19*:ti,ab,kw	333
#7	"2019-nCoV":ti,ab,kw	10
#8	SARS-CoV-2:ti,ab,kw	255
#9	SARS-CoV2:ti,ab,kw	60
#10	SARSCoV2:ti,ab,kw	209
#11	(Pneumonia AND Wuhan AND 2019):ti,ab,kw	94

#12 Coronavir*:ti,ab,kw 3624
#13 Coronovir*:ti,ab,kw 1
#14 HCov*:ti,ab,kw 17
#15 CV19*:ti,ab,kw 0
#16 CV-19*:ti,ab,kw 12
#17 N-Cov:ti,ab,kw 22
#18 #1 OR #2 OR #3 OR #4 OR #5 OR #6 OR #7 OR #8 OR #9 OR #10 OR #11 OR #12 OR #13 OR #14 OR #15 OR #16 OR #17 6352
#19 (Multisystem NEAR/2 Inflammatory):ti,ab,kw 29
#20 (Multi-System NEAR/2 Inflammatory):ti,ab,kw 13
#21 MISC:ti,ab,kw 59
#22 MIS-C:ti,ab,kw 3
#23 PIMS:ti,ab,kw 114
#24 #19 OR #20 OR #21 OR #22 OR #23 200
#25 #18 AND #24 5
#26 MeSH descriptor: [Americas] this term only 18
#27 MeSH descriptor: [Latin America] explode all trees 119
#28 (Latin NEAR/1 America*):ti,ab,kw 1332
#29 Latinamerica*:ti,ab,kw 70
#30 Latinoamerica*:ti,ab,kw 38
#31 Hispanoamerica*:ti,ab,kw 1
#32 Iberoamerica*:ti,ab,kw 14
#33 (Ibero NEAR/1 Americ*):ti,ab,kw 4
#34 Panamerican*:ti,ab,kw 10
#35 MeSH descriptor: [Central America] explode all trees 278
#36 Centroamerica*:ti,ab,kw 1
#37 Mesoamerica*:ti,ab,kw 5
#38 (Meso NEAR/1 America*):ti,ab,kw 0
#39 (Middle NEAR/1 America*):ti,ab,kw 29
#40 MeSH descriptor: [South America] explode all trees 2551
#41 (South NEAR/1 America*):ti,ab,kw 765
#42 Southamerica*:ti,ab,kw 8
#43 Sudamerica*:ti,ab,kw 5
#44 (America NEAR/2 Sur):ti,ab,kw 40
#45 MeSH descriptor: [Caribbean Region] explode all trees 403
#46 Caribbean:ti,ab,kw 559
#47 Caribe*:ti,ab,kw 30
#48 MeSH descriptor: [West Indies] explode all trees 385
#49 (West NEAR/1 Indi*):ti,ab,kw 75
#50 Antill*:ti,ab,kw 15
#51 MeSH descriptor: [Indians, South American] explode all trees 12

#52	MeSH descriptor: [Indians, South American] explode all trees	12
#53	Amerindian*:ti,ab,kw	16
#54	Indians:ti,ab,kw	634
#55	(American NEAR/1 Indian*):ti,ab,kw	584
#56	(Native NEAR/1 America*):ti,ab,kw	301
#57	Patagoni*:ti,ab,kw	1
#58	Andes:ti,ab,kw	2940
#59	Andean*:ti,ab,kw	51
#60	Amazon*:ti,ab,kw	379
#61	Argentin*:ti,ab,kw	962
#62	Bolivia*:ti,ab,kw	129
#63	Brazil*:ti,ab,kw	6146
#64	Brasil*:ti,ab,kw	549
#65	Colombia*:ti,ab,kw	1002
#66	Chile*:ti,ab,kw	869
#67	Ecuador*:ti,ab,kw	232
#68	Guiana*:ti,ab,kw	7
#69	Guyan*:ti,ab,kw	30
#70	Paraguay*:ti,ab,kw	41
#71	Peru*:ti,ab,kw	2052
#72	Surinam*:ti,ab,kw	48
#73	Uruguay*:ti,ab,kw	101
#74	Venez*:ti,ab,kw	210
#75	Belize*:ti,ab,kw	18
#76	(Costa NEAR/1 Ric*):ti,ab,kw	127
#77	Costaric*:ti,ab,kw	0
#78	Costarric*:ti,ab,kw	2
#79	Salvador*:ti,ab,kw	171
#80	Guatemal*:ti,ab,kw	355
#81	Hondur*:ti,ab,kw	104
#82	Nicaragu*:ti,ab,kw	87
#83	Panam*:ti,ab,kw	158
#84	MeSH descriptor: [Mexico] explode all trees	641
#85	Mexic*:ti,ab,kw	3115
#86	Cuba*:ti,ab,kw	490
#87	Dominic*:ti,ab,kw	146
#88	Haiti*:ti,ab,kw	234
#89	MeSH descriptor: [Puerto Rico] explode all trees	114
#90	(Puerto NEAR/1 Ric*):ti,ab,kw	467
#91	Puertorric*:ti,ab,kw	0
#92	Puertoric*:ti,ab,kw	6

#93 #26 OR #27 OR #28 OR #29 OR #30 OR #31 OR #32 OR #33 OR #34 OR #35 OR #36 OR #37 OR #38 OR #39 OR #40 OR #41 OR #42 OR #43 OR #44 OR #45 OR #46 OR #47 OR #48 OR #49 OR #50 OR #51 OR #52 OR #53 OR #54 OR #55 OR #56 OR #57 OR #58 OR #59 OR #60 OR #61 OR #62 OR #63 OR #64 OR #65 OR #66 OR #67 OR #68 OR #69 OR #70 OR #71 OR #72 OR #73 OR #74 OR #75 OR #76 OR #77 OR #78 OR #79 OR #80 OR #81 OR #82 OR #83 OR #84 OR #85 OR #86 OR #87 OR #88 OR #89 OR #90 OR #91 OR #92 21315

#94 #25 AND #93 0

CINAHL (EBSCO) 1/7/2021

#	Query	Results
S30	S26 AND S29 Limiters - Published Date: 20210501-20210731	5
S29	S27 OR S28	142,531
S28	AB (Latin N1 America*) OR Latinamerica* OR Latinoamerica* OR Latin* OR Hispanic Americans OR Iberoamerica* OR (Ibero N1 Americ*) OR Panamerican* OR (Central N1 America*) OR Centroamerica* OR Mesoamerica* OR (Meso N1 America*) OR (Middle N1 America*) OR (South N1 America*) OR Southamerica* OR Sudamerica* OR (America N1 Sur) OR Caribbean OR Caribe* OR (West N1 Indi*) OR Antill* OR Amerindian* OR Indians OR (American N1 Indian*) OR (Native N1 America*) OR Patagoni* OR Andes OR Andean* OR Amazon* OR Argentin* OR Bolivia* OR Brazil* OR Brasil* Colombia* OR Colombia* OR Colombia OR Chile* OR Ecuador* OR Guiana* OR Guyan* OR Guyan* OR Paraguay* OR Paraguay* OR Peru* OR Surinam* OR Surinam* OR Uruguay* OR Venez* OR Belize* OR (Costa N1 Ric*) OR Costarric* OR Costaric* OR Costa Ric* OR Costarric* OR Salvador* OR Salvador* OR Guatemal* OR Guatemal* OR Guatemala OR Hondur* OR Nicaragu* OR Panam* OR Mexic* OR Cuba* OR Dominic* OR Dominic*	142,531

	OR Haiti* OR Jamaic* OR (Puerto N1 Ric*) OR Puertorric* OR Puertoric*	
S27	TI (Latin N1 America*) OR Latinamerica* OR Latinoamerica* OR Latin* OR Hispanic Americans OR Iberoamerica* OR (Ibero N1 Americ*) OR Panamerican* OR (Central N1 America*) OR Centroamerica* OR Mesoamerica* OR (Meso N1 America*) OR (Middle N1 America*) OR (South N1 America*) OR Southamerica* OR Sudamerica* OR (America N1 Sur) OR Caribbean OR Caribe* OR (West N1 Indi*) OR Antill* OR Amerindian* OR Indians OR (American N1 Indian*) OR (Native N1 America*) OR Patagoni* OR Andes OR Andean* OR Amazon* OR Argentin* OR Bolivia* OR Brazil* OR Brasil* Colombia* OR Colombia* OR Colombia OR Chile* OR Ecuador* OR Guiana* OR Guyan* OR Guyan* OR Paraguay* OR Paraguay* OR Peru* OR Surinam* OR Surinam* OR Uruguay* OR Venez* OR Belize* OR (Costa N1 Ric*) OR Costarric* OR Costaric* OR Costa Ric* OR Costarric* OR Salvador* OR Salvador* OR Guatemal* OR Guatemal* OR Guatemala OR Hondur* OR Nicaragu* OR Panam* OR Mexic* OR Cuba* OR Dominic* OR Dominic* OR Haiti* OR Jamaic* OR (Puerto N1 Ric*) OR Puertorric* OR Puertoric*	142,531
S26	S19 AND S25	202
S25	S20 OR S21 OR S22 OR S23 OR S24	1,098
S24	TI PIMS OR AB PIMS	694
S23	TI MISC OR AB MISC	45
S22	TI MIS-C OR AB MIS-C	131
S21	TI (Multi-System N2 Inflammatory) OR AB (Multi-System N2 Inflammatory)	28
S20	TI (Multisystem N2 Inflammatory) OR AB (Multisystem N2 Inflammatory)	341

S19	S1 OR S2 OR S3 OR S4 OR S5 OR S6 OR S7 OR S8 OR S9 OR S10 OR S11 OR S12 OR S13 OR S14 OR S15 OR S16 OR S17 OR S18	36,041
S18	TI N-Cov* OR AB N-Cov*	4
S17	TI CV-19* OR AB CV-19*	53
S16	TI CV-19* OR AB CV-19*	53
S15	TI CV19* OR AB CV19*	1
S14	TI HCov* OR AB HCov*	85
S13	TI Coronovir* OR AB Coronovir*	8
S12	TI Coronavir* OR AB Coronavir*	14,038
S11	TI (Pneumonia AND Wuhan AND 2019) OR AB (Pneumonia AND Wuhan AND 2019)	217
S10	TI SARSCoV2 OR AB SARSCoV2	9
S9	TI SARS-CoV2 OR AB SARS-CoV2	195
S8	TI SARS-CoV-2 OR AB SARS-CoV-2	5,857
S7	TI 2019-nCoV OR AB 2019-nCoV	210
S6	TI COVID19* OR AB COVID19*	220
S5	TI COVID-19 OR AB COVID-19	30,675
S4	TI (Coron* N1 Virus) OR AB (Coron* N1 Virus)	417
S3	MH "COVID-19"	10,977
S2	(MH "Coronavirus Infections+")	17,989
S1	(MH "Coronavirus+")	1,211

LILACS (BVS-Eng) 1/7/2021

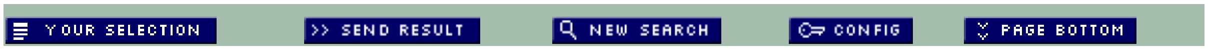

Database :	**LILACS**
Search on :	**(MH Coronavirus OR MH SARS-CoV-2 OR MH COVID-19 OR Corona OR COVID-19 OR COVID19$ OR 2019-nCoV OR SARS-CoV-2 OR SARS-CoV2 OR SARSCoV2 OR Coronavir$ OR Coronovir$ OR Corono OR HCov$ OR CV19$ OR CV-19$ OR N-Cov) AND (Multisystem$ OR Multi-System$ OR Multisistémico OR Multissistêmico OR MISC OR MIS-C OR PIMS OR SIMS OR SIMP OR SIM-P) [Words] and 2021 [Country, year publication]**
References found :	**15** [refine]
Displaying:	**1 .. 10** in format [**Detailed**]

2/7/2021 Global Health <1910 to 2021 Week 26>

1	exp Coronaviridae/	10441
2	(Coron* adj1 Virus).ti,ab.	778
3	COVID-19.ti,ab.	31746
4	COVID19*.ti,ab.	353
5	2019-nCoV.ti,ab.	1106
6	SARS-CoV-2.ti,ab.	13901
7	SARS-CoV2.ti,ab.	514
8	SARSCoV2.ti,ab.	13
9	(Pneumonia and Wuhan and "2019").ti,ab.	454
10	Coronavir*.ti,ab.	21299
11	Coronovir*.ti,ab.	12
12	HCov*.ti,ab.	556
13	CV19*.ti,ab.	8
14	CV-19*.ti,ab.	16
15	N-Cov.ti,ab.	8
16	or/1-15	41856
17	(Multisystem adj2 Inflammatory).ti,ab.	238
18	(Multi-System adj2 Inflammatory).ti,ab.	16
19	MISC.ti,ab.	87
20	MIS-C.ti,ab.	116

21 PIMS.ti,ab. 148

22 or/17-21 479

23 16 and 22 224

24 (Latin America* or Latinamerica* or Latinoamerica* or Latin* or Hispanic Americans or Iberoamerica* or Ibero Americ* or Panamerican* or Central America* or Centroamerica* or Mesoamerica* or Meso America* or Middle America* or South America* or Southamerica* or Sudamerica* or America del sur or Caribbean or Caribe* or West Indi* or Antill* or Amerindian* or Indians or American Indian* or Native America* or Patagoni* or Andes or Andean* or Amazon* or Argentin* or Bolivia* or Brazil* or Brasil* Colombia* or Colombia* or Colombia or Chile* or Ecuador* or Guiana* or Guyan* or Guyan* or Paraguay* or Paraguay* or Peru* or Surinam* or Surinam* or Uruguay* or Venez* or Belize* or Costa Ric* or Costarric* or Costaric* or Costa Ric* or Costarric* or Salvador* or Salvador* or Salvador or Guatemal* or Guatemal* or Guatemala or Hondur* or Nicaragu* Panam* or Mexic* or Cuba* or Dominic* or Dominic* or Haiti* or Jamaic* or Puerto Ric* or Puertorric* or Puertoric*).ti,ab. 228194

25 23 and 24 11

26 limit 25 to yr="2021 -Current" 4

EMBase (Elsevier) 4/7/2021

....

No.	Query Results	Results	Date
#29.	#28 AND 2021:py	11	
#28.	#26 AND #27	21	
#27.	americas:ti,ab OR 'south and central america'/exp OR ((latin NEAR/1 america*):ti,ab) OR latinamerica*:ti,ab OR latinoamerica*:ti,ab OR hispanoamerica:ti,ab OR iberoamerica*:ti,ab OR ((ibero NEAR/1 americ*):ti,ab) OR panamerica*:ti,ab OR ((south NEAR/1 america*):ti,ab) OR southamerica*:ti,ab OR sudamerica*:ti,ab OR (america:ti,ab AND del:ti,ab AND sur:ti,ab) OR ((central NEAR/1 america*):ti,ab) OR centroamerica*:ti,ab OR mesoamerica*:ti,ab OR ((meso NEAR/1 america*):ti,ab) OR ((middle NEAR/1 america*):ti,ab) OR 'caribbean'/exp OR 'caribbean islands'/exp OR caribbean*:ti,ab OR caribe*:ti,ab OR ((west NEAR/1 indi*):ti,ab) OR antill*:ti,ab OR 'american indian'/exp OR amerindian*:ti,ab OR indians:ti,ab OR ((native NEAR/1 america*):ti,ab)	555,976	

OR patagoni*:ti,ab OR andes:ti,ab OR andean*:ti,ab OR amazon*:ti,ab OR 'argentina'/exp OR argentin*:ti,ab OR 'bolivia'/exp OR bolivia*:ti,ab OR 'brazil'/exp OR brazil*:ti,ab OR brasil*:ti,ab OR 'colombia'/exp OR colombia*:ti,ab OR 'chile'/exp OR chile*:ti,ab OR 'ecuador'/exp OR ecuador*:ti,ab OR 'french guiana'/exp OR guiana*:ti,ab OR 'guyana'/exp OR guyan*:ti,ab OR 'paraguay'/exp OR paraguay*:ti,ab OR 'peru'/exp OR peru*:ti,ab OR 'suriname'/exp OR surinam*:ti,ab OR 'uruguay'/exp OR uruguay*:ti,ab OR 'venezuela'/exp OR venez*:ti,ab OR 'belize'/exp OR beliz*:ti,ab OR 'costa rica'/exp OR 'costa rica':ti,ab OR costarric*:ti,ab OR costaric*:ti,ab OR 'el salvador'/exp OR salvador*:ti,ab OR 'guatemala'/exp OR guatemal*:ti,ab OR 'honduras'/exp OR hondur*:ti,ab OR 'nicaragua'/exp OR nicaragu*:ti,ab OR 'panama'/exp OR panam*:ti,ab OR 'mexico'/exp OR mexic*:ti,ab OR mejic*:ti,ab OR 'cuba'/exp OR cuba*:ti,ab OR 'dominican republic'/exp OR dominica*:ti,ab OR 'haiti'/exp OR haiti*:ti,ab OR 'jamaica'/exp OR jamaic*:ti,ab OR 'puerto rico'/exp OR ((puerto NEAR/1 ric*):ti,ab) OR puertoric*:ti,ab OR puertorric*:ti,ab

#26. #19 AND #25 594

#25. #21 OR #22 OR #23 OR #24 2,934

#24. pims:ti,ab 1,990

#23. 'mis c':ti,ab 491

#22. misc:ti,ab 319

#21. ('multi system' NEAR/2 inflammatory):ti,ab 203

#20. (multisystem NEAR/2 inflammatory):ti,ab 1,871

#19. #1 OR #2 OR #3 OR #4 OR #5 OR #6 OR #7 OR #8 OR #9 OR #10 OR #11 OR #12 OR #13 OR #14 OR #15 OR #16 OR #17 OR #18 178,865

#18. 'n cov':ti,ab 29

#17. 'cv 19*':ti,ab 128

#16. cv19*:ti,ab 24

#15. hcov*:ti,ab 1,096

#14. coronovir*:ti,ab 61

#13.	coronavir*:ti,ab	63,544
#12.	pneumonia:ti,ab AND wuhan:ti,ab AND 2019:ti,ab	1,161
#11.	'sarscov2':ti,ab	97
#10.	'sars cov2':ti,ab	2,034
#9.	'sars cov 2':ti,ab	40,987
#8.	'2019 ncov':ti,ab	1,307
#7.	covid19*:ti,ab	1,938
#6.	'covid 19':ti,ab	127,107
#5.	(coron* NEAR/1 virus):ti,ab	2,350
#4.	'coronavirus disease 2019'/exp	127,157
#3.	'severe acute respiratory syndrome coronavirus 2'/exp	35,276
#2.	'coronavirinae'/exp	62,765
#1.	'pediatric multisystem inflammatory syndrome'/exp	567
